# Bradycardia, Renal Dysfunction, Atrioventricular Node Blockade, Shock, and Hyperkalemia (BRASH) Syndrome: Clinical Features, Outcomes, and Therapeutic Implications

**DOI:** 10.7759/cureus.101875

**Published:** 2026-01-19

**Authors:** Mariana Esteves, Rita Bragança, Sandra Morais

**Affiliations:** 1 Internal Medicine, Unidade Local de Saúde de Trás-os-Montes e Alto Douro, Vila Real, PRT; 2 Internal Medicine, Unidade Local de Saúde de Trás-Os-Montes e Alto Douro, Vila Real, PRT

**Keywords:** acute kidney injury, atrioventricular node blockade, bradycardia, brash syndrome, elderly patients, hyperkalemia, polypharmacy, shock

## Abstract

Introduction: BRASH syndrome, characterized by the combination of bradycardia, renal dysfunction, atrioventricular node-blocking agent use, shock, and hyperkalemia, is an increasingly recognized but underdiagnosed clinical pattern, particularly in elderly patients with multiple comorbidities. Data regarding its clinical presentation, management, and short-term outcomes remain limited.

Methods: We conducted a single-center retrospective cohort study including patients admitted to the emergency department between 2019 and 2022. Patients met clinical criteria including bradycardia, hyperkalemia (K+≥5.0 mmol/L), acute kidney injury (KDIGO) or acute-on-CKD, AV-nodal blocker use, and evidence of shock. Demographic characteristics, comorbidities, precipitating factors, treatments, and short-term outcomes were analyzed. Subgroup analyses were exploratory.

Results: Fifty-one patients were included. The mean age was 79.8 years (SD 10.6; range 48-102), with a balanced sex distribution. Hypertension (90.2%), heart failure (68.6%), atrial fibrillation (52.9%), and chronic kidney disease (25.5%) were common. Most patients were receiving beta-blockers (82.4%), angiotensin-converting enzyme inhibitors (68.6%), calcium channel blockers (37.3%), or digoxin (31.4%). Frequent precipitating factors included nephrotoxic exposure (49.0%), infection or sepsis (43.1%), dose initiation or escalation of atrioventricular node-blocking agents (43.1%), and hypovolemia (33.3%). Admission hyperkalemia was generally mild to moderate, with a median potassium level of 6.5 mmol/L (IQR 5.8-7.2). Management included intravenous calcium (72.5%), insulin with dextrose (72.5%), beta-agonists (88.2%), vasopressor support (33.3%), renal replacement therapy (11.8%), and pacing support (9.8%). In-hospital mortality was 15.7%. Post-discharge mortality was 2.3% at 30 days and 7.0% at 90 days, and hospital readmission within 90 days occurred in 41.9%.

Conclusions: Early recognition of BRASH syndrome is essential, as even moderate hyperkalemia may lead to significant hemodynamic compromise and organ support requirements in high-risk patients.

## Introduction

Bradycardia, renal failure, atrioventricular nodal blockade, shock, and hyperkalemia (BRASH) syndrome is a recently recognized clinical entity characterized by the synergistic interaction of five interdependent components: bradycardia, renal dysfunction, atrioventricular (AV) nodal blockade, shock, and hyperkalemia [1]. First formally described as a distinct syndrome in 2016, BRASH represents a self-reinforcing cycle, rather than a coincidental cluster of unrelated abnormalities [1,2].

The pathophysiology of BRASH syndrome is driven by the combined effects of AV nodal-blocking agents and hyperkalemia, which together exert a disproportionate negative chronotropic and dromotropic effect [1,3]. Even mild to moderate elevations in serum potassium may significantly potentiate AV nodal suppression in patients receiving beta-blockers, non-dihydropyridine calcium channel blockers, digoxin, or other AV nodal-modulating medications [1,3]. The resulting bradycardia reduces cardiac output and leads to systemic hypoperfusion. Renal function subsequently worsens, impairing the clearance of potassium and AV nodal-blocking drugs. This process further amplifies hyperkalemia and bradycardia, creating a malignant feedback loop [2,4].

A defining clinical feature of BRASH syndrome is the presence of severe bradycardia that appears disproportionate to the degree of hyperkalemia and frequently occurs in the absence of the classic electrocardiographic changes typically associated with isolated hyperkalemia [3,5]. This dissociation often leads to diagnostic error, with clinicians attributing the presentation to isolated drug toxicity, primary conduction disease, or electrolyte disturbance rather than recognizing the underlying syndrome [2,6]. Consequently, standard advanced cardiac life support algorithms for bradycardia may be ineffective unless the underlying pathophysiological cycle of BRASH syndrome is addressed in a comprehensive manner [6,7].

BRASH syndrome predominantly affects elderly patients with underlying cardiovascular disease and chronic kidney disease, particularly in the setting of polypharmacy [4,8]. Minor physiological stressors, including dehydration, infection, gastrointestinal illness, or modest medication adjustments, may be sufficient to precipitate the syndrome. This is particularly true in patients with limited renal or cardiac reserve [9,10]. These seemingly minor insults can trigger rapid clinical deterioration, highlighting the vulnerability of this population.

Although the true epidemiology of BRASH syndrome remains incompletely defined, accumulating evidence suggests that it is underrecognized and more prevalent than previously appreciated [4,8]. Recent observational data indicate that BRASH syndrome and related pre-BRASH states may account for a clinically meaningful proportion of hospital admissions for symptomatic bradycardia and are associated with increased in-hospital mortality compared with isolated bradyarrhythmias [11]. Systematic reviews and contemporary case series describe a broad clinical spectrum, ranging from mild, reversible presentations to severe cases requiring renal replacement therapy, vasoactive support, or temporary cardiac pacing [4,8,12-15].

Early identification of BRASH syndrome is critical, as timely correction of hyperkalemia, discontinuation of offending agents, optimization of volume status, and appropriate hemodynamic support can often reverse the condition and prevent unnecessary invasive interventions [5,7,13]. Given its diagnostic challenges, variable clinical severity, and potential for rapid progression, increased clinical awareness of BRASH syndrome is essential for clinicians managing patients with unexplained bradycardia in the context of renal dysfunction and AV nodal blockade. The objective of this study was to descriptively characterize the clinical features, precipitating factors, management strategies, and short-term outcomes of patients presenting with a clinical pattern consistent with BRASH syndrome.

## Materials and methods

This was a single-center retrospective observational study conducted at a tertiary care hospital. Adult patients admitted through the emergency department between January 2019 and December 2022 were eligible for screening. The study was approved by the Ethics Committee of the Trás-os-Montes e Alto Douro Local Health Unit (approval number: 4729), with a waiver of informed consent due to the retrospective design and use of anonymized data.

All emergency department admissions in which the primary reason for presentation was a documented heart rate disturbance were initially reviewed. These included patients admitted with either bradycardia or tachycardia as the main presenting problem. A total of 725 patients met this broad screening criterion. Individual medical records were then reviewed to identify patients with bradycardia as the predominant clinical feature during admission. Bradycardia was defined as a heart rate below 60 beats per minute. After this step, 370 patients remained eligible for further assessment. From this bradycardic population, patients were included in the final cohort if they fulfilled all predefined criteria for the BRASH clinical pattern during the same admission: (i) bradycardia; (ii) hyperkalemia, defined as a serum potassium level ≥5.0 mmol/L at presentation; (iii) acute kidney injury or acute-on-chronic kidney disease; (iv) current treatment with at least one AV node-blocking agent; and (v) hemodynamic instability compatible with shock, consistent with previously published definitions of BRASH syndrome [1]. All laboratory values, electrocardiographic findings, and vital signs used to define eligibility were obtained within the first hour of emergency department presentation, using the earliest documented measurements. Patients with missing critical data (e.g., potassium, creatinine, or medication history) were excluded from the final analysis.

Acute kidney injury was defined according to the Kidney Disease: Improving Global Outcomes (KDIGO) criteria, based on changes in serum creatinine relative to baseline values when available [16]. Acute-on-chronic kidney disease was defined as acute kidney injury occurring in patients with previously documented chronic kidney disease, with CKD staging based on KDIGO clinical practice guidelines [17]. Shock was defined as a systolic blood pressure below 90 mmHg and/or the requirement for vasopressor support during admission, in accordance with established clinical definitions [18]. High-grade AV block was defined as second-degree AV block, Mobitz type II or third-degree (complete) AV block, according to established electrophysiological definitions and international guidelines [19]. Hyperkalemia was assessed using the first serum potassium measurement obtained at hospital presentation. Baseline laboratory values were retrieved when available to assess changes in renal function. Data collected included demographic characteristics, comorbidities, chronic medications, potential precipitating factors, laboratory findings, electrocardiographic features, therapeutic interventions, and clinical outcomes. Precipitating factors were identified by structured chart review of ED and inpatient notes, medication lists, microbiology results, imaging, and discharge diagnoses. Factors were categorized a priori (infection/sepsis, hypovolemia, nephrotoxic exposure, heart failure decompensation, and AV nodal blocker initiation/escalation). When documentation was ambiguous, cases were adjudicated by consensus between two investigators.

The primary outcomes of interest were in-hospital mortality, 30-day, and 90-day post-discharge mortality. Secondary outcomes included need for temporary or permanent pacing, intensive care unit admission, length of hospital stay, and hospital readmission.

Statistical analyses were exploratory and primarily descriptive, as the study was not powered to detect intergroup differences. Continuous variables were assessed for distribution and are presented as medians with ranges or interquartile ranges, as appropriate. Categorical variables are presented as counts and percentages. Comparisons between groups were performed using non-parametric tests for continuous variables and chi-square or Fisher’s exact test for categorical variables, as appropriate. A two-sided p-value <0.05 was considered statistically significant. Statistical analyses were performed using IBM SPSS Statistics for Windows, Version 29 (Released 2023; IBM Corp., Armonk, New York, United States). No clinical scoring systems, questionnaires, or proprietary assessment tools were used in this study.

## Results

Patient selection

**Figure 1:**
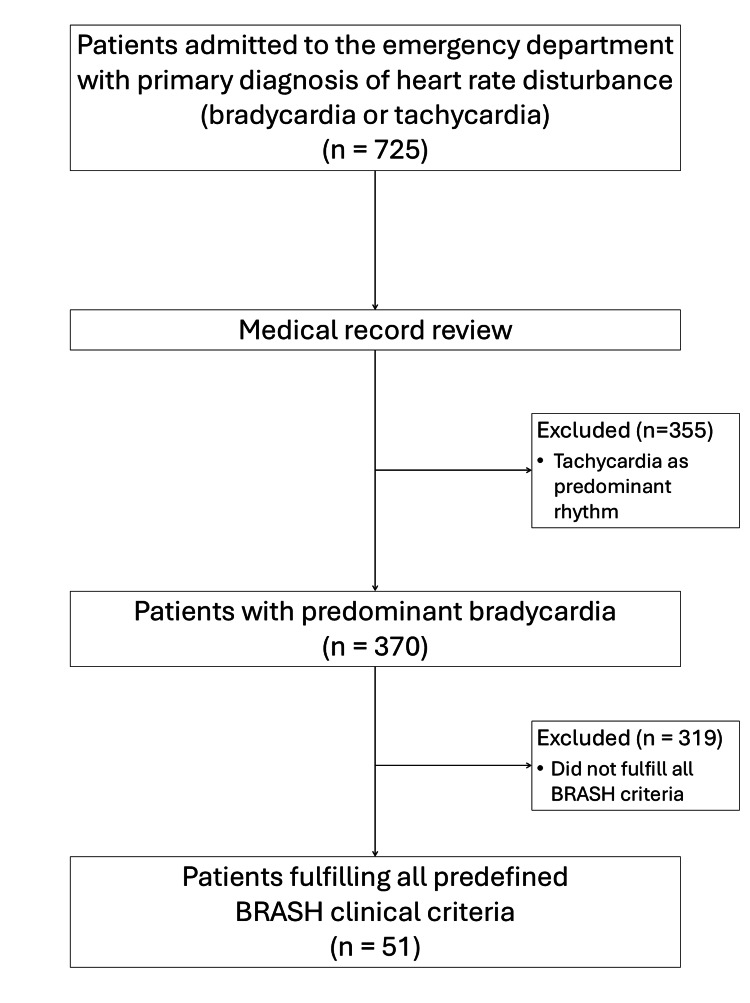
Flow diagram of patient selection During the study period, 725 patients were admitted to the emergency department with a primary diagnosis of heart rate disturbance (bradycardia or tachycardia). After individual medical record review, patients with tachycardia as the predominant rhythm were excluded, resulting in 370 patients with predominant bradycardia. Of these, 319 patients did not fulfil all predefined BRASH clinical criteria. The final study cohort comprised 51 patients. BRASH: bradycardia, renal failure, atrioventricular nodal blockade, shock, and hyperkalemia

During the study period, 725 patients were admitted to the emergency department with a primary diagnosis of heart rate disturbance, including bradycardia or tachycardia. After individual medical record review, 370 patients were identified in whom bradycardia was the predominant clinical feature. Among these, 51 patients fulfilled all predefined criteria for the BRASH clinical pattern and were included in the final cohort (Figure 1).

Baseline characteristics

The cohort consisted predominantly of very elderly patients with a high burden of cardiovascular comorbidities. The mean age at admission was 79.8 ± 10.6 years (range 48-102), and 51% of patients were female. Hypertension (90.2%), heart failure (68.6%), atrial fibrillation (52.9%), and diabetes mellitus (43.1%) were highly prevalent.

Chronic kidney disease was present in 13 patients (25.5%), encompassing CKD stages 2 to 5. Of these, two patients had stage 2 CKD, one stage 3a, two stage 3b, four stage 4, and four stage 5 disease, with two patients receiving chronic maintenance hemodialysis prior to admission. Ischemic heart disease (25.5%), chronic obstructive pulmonary disease (19.6%), cirrhosis (5.9%), and sleep apnea (5.9%) were less frequent.

Most patients were receiving chronic AV node-blocking therapy prior to admission. Beta-blockers were the most commonly prescribed agents (82.4%), followed by angiotensin-converting enzyme inhibitors (68.6%), calcium channel blockers (37.3%), digoxin (31.4%), angiotensin receptor blockers (15.7%), and amiodarone (9.8%). Baseline demographic characteristics, comorbidities, and chronic medication use are summarized in Table 1.

**Table 1:** Baseline characteristics of patients admitted with BRASH syndrome SD: standard deviation; ACE: angiotensin-converting enzyme; ARB: angiotensin receptor blocker; CKD: chronic kidney disease; COPD: chronic obstructive pulmonary disease; BRASH: bradycardia, renal failure, atrioventricular nodal blockade, shock, and hyperkalemia CKD stages were defined according to the Kidney Disease: Improving Global Outcomes (KDIGO) 2024 clinical practice guideline [17].

Baseline characteristics	
Demographic	
Age at admission, mean ± SD	79.8 ± 10.6
Female sex, n (%)	26 (51.0%)
Comorbidities, n (%)	
Hypertension	46 (90.2%)
Heart failure	35 (68.6%)
Atrial fibrillation	27 (52.9%)
Diabetes mellitus	22 (43.1%)
Chronic kidney disease	13 (25.5%)
CKD stage 2	2 (3.9%)
CKD stage 3a	1 (2.0%)
CKD stage 3b	2 (3.9%)
CKD stage 4	4 (7.8%)
CKD stage 5	4 (7.8%)
Ischemic heart disease	13 (25.5%)
COPD	10 (19.6%)
Cirrhosis	3 (5.9%)
Sleep apnea	3 (5.9%)
Peripheral arterial disease	1 (2.0%)
Medication, n (%)	
Diuretics	47 (92.2%)
Beta-blockers	42 (82.4%)
ACE inhibitors	35 (68.6%)
Calcium channel blockers	19 (37.3%)
Digoxin	16 (31.4%)
ARBs	8 (15.7%)
Amiodarone	5 (9.8%)
Potassium-binding resins	1 (2.0%)

Clinical presentation, laboratory findings, and management

All patients presented with bradycardia by definition. Common presenting symptoms included dyspnea (45.1%), fatigue or weakness (45.1%), altered consciousness (35.3%), chest pain (29.4%), dizziness (27.5%), syncope (11.8%), and vomiting (11.8%). Seizures were uncommon (3.9%), and cardiac arrest at presentation occurred in only one patient (2.0%).

Identifiable precipitating factors were frequent. The most common were nephrotoxic exposure (49.0%), infection or sepsis (43.1%), hypovolemia (33.3%), and acute or decompensated heart failure (33.3%). Initiation or dose escalation of an AV node-blocking agent was observed in 43.1% of patients. Admission characteristics, presenting symptoms, and precipitating factors are detailed in Table 2.

**Table 2:** Admission characteristics, presenting symptoms, and identified precipitating factors among patients with BRASH syndrome. AV: atrioventricular

Admission characteristics	
Admitting service, n (%)	
Internal Medicine	47 (92.2%)
Cardiology	2 (3.9%)
Other	2 (3.9%)
Presenting symptoms, n (%)	
Bradycardia	51 (100%)
Dyspnea	23 (45.1%)
Fatigue/weakness	23 (45.1%)
Altered consciousness	18 (35.3%)
Chest pain	15 (29.4%)
Dizziness	14 (27.5%)
Syncope	6 (11.8%)
Vomiting	6 (11.8%)
Seizure	2 (3.9%)
Cardiac arrest	1 (2.0%)
Trigger, n (%)	
Nephrotoxic exposure	25 (49.0%)
Infection/sepsis	22 (43.1%)
Hypovolemia	17 (33.3%)
Acute HF decompensation	17 (33.3%)
New AV-node blocker	12 (23.5%)
Dose escalation of AV-node blocker	10 (19.6%)

Hyperkalemia at admission was generally mild to moderate, with a median potassium level of 6.5 mmol/L (IQR 5.8-7.2). Compared with baseline values, serum potassium increased by approximately 2.0 mmol/L at presentation. Acute kidney injury was present in all patients, occurring either in individuals without baseline chronic kidney disease or as acute deterioration in those with pre-existing chronic kidney disease. Median serum creatinine increased from 1.2 mg/dL at baseline to 2.6 mg/dL at admission, while median urea rose from 52 mg/dL to 130 mg/dL.

Classic electrocardiographic manifestations of hyperkalemia were uncommon. Peaked T waves were observed in eight patients (15.7%), QRS widening (>120 ms) in 11 patients (21.6%), and junctional rhythm in nine patients (17.6%). High-grade AV block was present in 14 patients (27.5%) and ST-segment deviations in five patients (9.8%).

Most patients received standard medical therapy for hyperkalemia. Insulin with dextrose was administered in 37 patients (72.5%), intravenous calcium salts in 72.5%, and beta-agonists in 88.2%. Additional therapies included bicarbonate administration (19.6%) and potassium-binding resins (82.4%). Atropine was administered in 39.2% of patients but was frequently ineffective. Vasopressor or chronotropic support was required in 33.3% of patients, and intravenous fluid resuscitation was administered in 70.6%.

Clinical outcomes

In-hospital mortality occurred in eight patients (15.7%). Among the 43 patients discharged alive, post-discharge mortality was observed in one patient (2.3%) at 30 days and in three patients (7.0%) at 90 days.

Permanent pacemaker implantation was required in five patients (9.8%), including four patients with high-grade AV block, while external pacing was used in two patients (3.9%), both of whom had high-grade AV block, during the index hospitalization. Renal replacement therapy was required in six patients (11.8%); of these, four patients required acute initiation of dialysis, whereas two patients were already receiving chronic maintenance hemodialysis prior to admission.

The median length of hospital stay was six days (IQR: 4-10). A total of 18 patients (41.9%) were readmitted within 90 days of discharge. In-hospital and post-discharge outcomes are summarized in Table 3.

**Table 3:** In-hospital outcomes and post-discharge follow-up of patients with BRASH syndrome ICU: intensive care unit; IQR: interquartile range; BRASH: bradycardia, renal failure, atrioventricular nodal blockade, shock, and hyperkalemia

Outcomes	
In-hospital outcomes, n (%)	
In-hospital mortality	8 (15.7%)
Length of hospital stay, days, median (IQR)	6 (4-10)
ICU admission	5 (9.8%)
Cardiac interventions during hospitalization, n (%)	
Permanent pacemaker implantation	5 (9.8%)
External pacing	2 (3.9%)
Renal outcomes during hospitalization, n (%)	
Renal replacement therapy (total)	6 (11.8%)
Acute initiation of dialysis	4 (7.8%)
Chronic hemodialysis prior to admission	2 (3.9%)
Post-discharge outcomes, n (%)	
30-day post-discharge mortality	1 (2.3%)
90-day post-discharge mortality	3 (7.0%)
Hospital readmission within 90 days	18 (41.9%)

Exploratory subgroup analyses

Exploratory analyses comparing clinically relevant subgroups are presented in Table 4.

**Table 4:** Exploratory comparisons between key clinical subgroups AKI: acute kidney injury; AV: atrioventricular; bpm: beats per minute; CKD: chronic kidney disease; IQR: interquartile range; OR: odds ratio; U: Mann–Whitney U statistic; z: standardized test statistic. High-grade atrioventricular block is defined as second-degree Mobitz type II or third-degree atrioventricular block, according to international electrophysiology guidelines [19]. AKI was defined according to the Kidney Disease: Improving Global Outcomes (KDIGO) criteria [16].

Exploratory comparisons of clinically relevant subgroups			
In-hospital mortality			
	Survivors (n = 43)	Non-survivors (n = 8)	p-value
Age, years, median	82	87	0.048 (Mann–Whitney U=248.3; z=1.98)
Admission potassium, mmol/L, median	5.6	5.7	0.81 (Mann–Whitney U=181.3; z=0.24)
Vasopressor requirement, n (%)	9 (20.9)	6 (75.0)	0.004 (Fisher exact, OR=0.09)
Pacing requirement			
	No pacing (n = 44)	Pacing required (n = 7)	p-value
Admission heart rate, bpm, median	38	30	0.01 (Mann–Whitney U=248.1; z=2.58)
High-grade atrioventricular block, n (%)	8 (18.2)	6 (85.7)	<0.001 (Fisher exact, OR=0.04)
≥2 AV-node blockers, n (%)	11 (25.0)	4 (57.1)	0.11 (Fisher exact, OR=0.25)
AKI vs acute-on-chronic kidney disease			
	AKI (n = 38)	Acute-on-CKD (n = 13)	p-value
Admission potassium, mmol/L, median	6.4 (5.6–7.1)	5.8	0.60 (Mann–Whitney U=271.3; z=0.52)
Renal replacement therapy, n (%)	2 (5.3)	4 (30.8)	0.031 (Fisher exact, OR=0.13)
Length of stay, days, median (IQR)	6 (2–11)	5 (2–7)	0.76 (Mann–Whitney U=261.1; z=0.31)

Patients who died during hospitalization were older and more frequently required vasopressor support compared with survivors, while admission potassium levels did not differ significantly between groups. Patients requiring pacing support more often presented with lower admission heart rates and advanced AV block on electrocardiography. When stratified by baseline renal function, patients with acute kidney injury due to chronic kidney disease more frequently required renal replacement therapy during hospitalization compared with those without baseline chronic kidney disease. No significant differences in admission potassium levels or length of hospital stay were observed between these renal subgroups. In an exploratory subset analysis, admission potassium levels were not significantly associated with intensive care unit admission (median 7.3 vs. 6.4 mmol/L for ICU vs. non-ICU patients; Mann-Whitney U test, p = 0.32). All subgroup analyses were exploratory and should be interpreted with caution.

## Discussion

This retrospective single-center cohort describes the clinical characteristics, precipitating factors, management, and short-term outcomes of patients presenting with a clinical pattern consistent with BRASH syndrome. By systematically analyzing a relatively large series compared with most previously published reports, this study contributes additional descriptive data to a still limited body of literature on this emerging clinical constellation.

Clinical profile and presentation

The patients included in this cohort were predominantly very elderly and had a high burden of cardiovascular comorbidities, particularly atrial fibrillation, with a substantial proportion also having chronic kidney disease and widespread use of AV node-blocking agents. This clinical profile is consistent with previous descriptions of BRASH syndrome, which have highlighted advanced age, impaired renal function, and polypharmacy as key predisposing factors [1,4]. Importantly, although hyperkalemia was present in all patients by definition, potassium levels at admission were generally mild to moderate. This finding supports the concept that BRASH syndrome represents a synergistic interaction between renal dysfunction, AV node blockade, and electrolyte disturbances rather than the effects of severe hyperkalemia alone [1,2].

Clinical presentation was heterogeneous and often non-specific, with dyspnea, weakness, altered mental status, and dizziness frequently observed. Such variability may contribute to under-recognition in the emergency department, where bradycardia or renal dysfunction may initially be attributed to a single cause rather than a multifactorial process. The frequent identification of precipitating factors such as infection, hypovolemia, acute heart failure decompensation, and nephrotoxic exposure underscores the dynamic nature of this syndrome and highlights potentially reversible contributors [3,8,10]. These findings emphasize that BRASH syndrome often arises from a convergence of chronic vulnerability and acute stressors, highlighting the importance of early identification and correction of reversible precipitants, alongside metabolic and hemodynamic stabilization.

Management and in-hospital course

Most patients were managed with standard medical therapy for hyperkalemia, while pacing support was required in a minority. The need for permanent pacemaker implantation or temporary external pacing was more frequently observed in patients with advanced AV block and profound bradycardia rather than being related to potassium levels themselves. This observation aligns with proposed pathophysiological mechanisms of BRASH syndrome, in which AV node blockade and renal dysfunction reinforce each other, leading to conduction disturbances that may be disproportionate to the degree of hyperkalemia [1].

Renal replacement therapy was required in a subset of patients, predominantly among those with baseline chronic kidney disease. In contrast, patients without baseline chronic kidney disease rarely required dialysis. This suggests that baseline renal reserve plays an important role in determining disease severity and clinical trajectory in this population.

Outcomes and exploratory analyses

In-hospital mortality in this cohort was considerable, reflecting the severity of illness at presentation and the high degree of comorbidity among affected patients. Although post-discharge mortality was lower, it was not negligible, emphasizing the ongoing vulnerability of this population even after apparent clinical stabilization. Readmission rates were also substantial, highlighting the need for careful post-discharge follow-up, medication review, and optimization of renal and cardiovascular management. In published BRASH cohorts and systematic reviews, both in-hospital mortality and the need for renal replacement therapy have been reported as clinically relevant outcomes, although reported rates vary substantially due to heterogeneity and publication bias [4,8,11].

Exploratory subgroup analyses provided additional descriptive insights. Patients who died during hospitalization were older and more frequently required vasopressor support, suggesting that hemodynamic instability may identify a particularly high-risk subgroup. Similarly, pacing requirement was strongly associated with advanced AV block and lower admission heart rates. Patients with acute-on-chronic kidney disease more frequently required renal replacement therapy, while the length of hospital stay did not differ significantly between groups. These findings should be interpreted cautiously and viewed as hypothesis-generating rather than indicative of causal relationships.

Positioning within the existing literature

Most existing literature on BRASH syndrome consists of isolated case reports and small case series. By describing a larger cohort over several years, this study adds incremental evidence regarding the clinical spectrum, common triggers, and short-term outcomes associated with this syndrome. While the present findings do not establish BRASH syndrome as a distinct pathophysiological entity, they support its utility as a pragmatic clinical framework that may facilitate earlier recognition and more integrated management in vulnerable patients [1,4].

Limitations

This study has several limitations. Its retrospective design and single-center setting limit generalizability. Case identification relied on predefined clinical criteria and manual chart review, introducing potential selection and misclassification bias. The absence of a comparator group precludes definitive conclusions regarding causality or the independent contribution of specific medications or precipitating factors. Statistical analyses were exploratory, and the small number of outcome events limits the robustness of subgroup comparisons. Management strategies were not standardized and reflected real-world clinician judgment, which may have introduced treatment heterogeneity. In addition, outcomes beyond 90 days could not be systematically assessed, limiting conclusions regarding long-term prognosis.

Clinical implications and future directions

Despite these limitations, this study highlights BRASH syndrome as a clinically relevant and potentially under-recognized pattern associated with significant morbidity and mortality. From a frontline perspective, these findings support closer electrolyte and renal function monitoring in elderly patients with chronic kidney disease receiving AV nodal-blocking agents, particularly during intercurrent illness or dehydration. In patients presenting with unexplained bradycardia and renal dysfunction, clinicians should maintain a low threshold to suspect BRASH even when hyperkalemia is modest and prioritize simultaneous reversal of hyperkalemia and withdrawal of AV nodal blockers. In unstable patients, early empiric intravenous calcium may be considered to mitigate membrane excitability while definitive correction is initiated [1-3,10].

Increased awareness of this constellation may prompt earlier identification, avoidance of reflexive escalation of AV node-blocking therapies, and more comprehensive evaluation of renal function and reversible triggers. Future prospective studies with comparator groups and standardized definitions are needed to better delineate prognostic factors and inform optimal management strategies.

## Conclusions

In this single-center retrospective cohort, BRASH syndrome predominantly affected elderly patients with multiple cardiovascular comorbidities and widespread use of AV node-blocking agents. Despite generally mild to moderate hyperkalemia, the syndrome was associated with substantial in-hospital mortality and frequent need for advanced supportive therapies.

These findings emphasize the importance of recognizing BRASH syndrome as a distinct and clinically relevant pattern in patients presenting with bradycardia and renal dysfunction. Early identification and prompt, integrated management targeting reversible precipitants and metabolic derangements may help mitigate disease severity and avoid unnecessary invasive interventions.
